# Comparison of Thermal and High-Pressure Gelation of Potato Protein Isolates

**DOI:** 10.3390/foods9081041

**Published:** 2020-08-02

**Authors:** Hadas Katzav, Libi Chirug, Zoya Okun, Maya Davidovich-Pinhas, Avi Shpigelman

**Affiliations:** Faculty of Biotechnology and Food Engineering and Russell Berrie Nanotechnology Institute, Technion, Israel Institute of Technology, Haifa 3200003, Israel; hadas.katzav@gmail.com (H.K.); chiruglibi@walla.co.il (L.C.); zoya@bfe.technion.ac.il (Z.O.); dmaya@bfe.technion.ac.il (M.D.-P.)

**Keywords:** alternative protein, high hydrostatic pressure, high-pressure gelation, protein gels, thermal gelation

## Abstract

Potato protein isolate (PPI), a commercial by-product of the starch industry, is a promising novel protein for food applications with limited information regarding its techno-functionality. This research focused on the formation of both thermal and high-pressure gels at acidic and neutral pH levels. Our results reveal that physical gels are formed after 30 min by heat at pH 7 and pH 3, while pressure (300–500 MPa) allows the formation of physical gels only at pH 3, and only when the system crosses 30 °C by adiabatic heating during pressurization. Texture profile analysis (TPA) revealed that gel hardness increased with both gelation temperature and pressure, while water-holding capacity was lower for the pressure-induced gels. The proteins released in the water-holding test suggested only partial involvement of patatin in the gel formation. Vitamin C as a model for a thermally liable compound verified the expected better conservation of such compounds in a pressure-induced gel compared to a thermal one of similar textural properties, presenting a possible advantage for pressure-induced gelation.

## 1. Introduction

In recent years, there is a growing interest in replacing (at least partially) animal proteins with plant proteins in various foods applications [1,2]. Plant-based protein ingredients have gained a lot of attention due to their suggested contribution to sustainability and food security challenges, in addition to their cost-effectiveness (in some cases) in comparison to animal-based proteins. However, utilization of plant proteins is often insufficiently studied, and commercially limited, partially as a result of their techno-functional properties (such as poor aqueous solubility), anti-nutritional components, off-flavor, and color [3].

Legumes, oilseeds and cereals are widely discussed as plant-based sources for proteins, while studies regarding fruits and vegetables as protein sources are less common [4], mostly due to the relatively low protein content in those sources. Potato has a relatively low protein concentration of about 1.7% wet base, although potatoes are the second-highest protein providing crops per hectare grown after wheat [5]. Potato protein is a product of thermal and acidic coagulation of potato juice obtained during starch production, therefore, it can be considered as a novel protein source, and can help overcome the economic impact related to its high polluting capacity [5,6,7,8]. Potato protein was reported to contain the essential amino acid composition and suggested to be nutritionally equivalent to animal protein [5]. The potato proteins main groups, patatin, protease inhibitors, and high-molecular-weight proteins, were suggested to be associated with high nutritional value, ability to regulate serum cholesterol level, antioxidant potential, antimicrobial effects, and lower allergic response compared to other proteins [5,9]. Potato proteins were reported to unfold between 55 °C–75 °C with increasing denaturation temperature as a function of increasing ionic strength [10]. Patatin (or tuberin) is a group of glycoproteins that are the major constituent of potato protein isolate (~40%), with a molecular weight of 45–50 kDa and an isoelectric point at pH = 4.9 [5] or 5.25 [11], and was found to be stable at least up to 45 °C. Protease inhibitors have a range of molecular weights of 5 kDa to 25 kDa, they are more hydrophilic than patatin, and tend to coagulate by heat [5].

Protein functional properties of importance for food processing include solubility, water and oil binding, emulsification, foaming, gelation, and thickening [12]. Protein gelation is an important characteristic, aiming to stabilize food structure, resulting in desirable sensory and textural attributes in food products. The protein gelation requires a driving force to unfold the native protein structure, which is followed by an aggregation phenomenon that retains some degree of order in the matrix formed by interactions between the protein strands [13]. Protein gelation is a two-step process of denaturation and aggregation and can be classified by gelation induced using physical means (heat and pressure) or chemical means (acidification, enzymatic cross-linking, use of salts and urea) [13,14]. The characteristics of the formed gel depend on factors such as protein concentration, degree of denaturation (as affected by pH), temperature, ionic strength, and pressure [13,14]. In heat-induced gelation, an increase in the effective hydrophobicity is an indication of protein unfolding, and when sufficient hydrophobic sites are exposed, interactions will occur between the exposed hydrophobic sites, resulting in protein aggregation [13]. The total time of heating and the rate of temperature increase also affects protein unfolding. A recent review concluded that the general features of gels from plant globulins are similar to gels of globular proteins from animal sources [15]. However, protein gels from animal origin, such as whey proteins, were often found to be stiffer and require a lower protein concentration (for gel formation) compared to studied plant protein gels (soy and pea) [14]. Additionally, it was suggested that the protein extraction protocol can have a major impact on the properties of heat-set plant globulins gels [15]. While information regarding thermal gelation of plant proteins exists regarding cereals, oilseeds, pulses, and legumes protein, the information regarding gelation of fruit and vegetable proteins is extremely scarce [4].

High pressure processing (HPP) or high hydrostatic pressure (HHP) was developed with the main purpose of obtaining microbiologically safe food products [16], while avoiding/minimizing undesirable changes in the sensorial, physicochemical, and nutritional properties. HHP enables transmittance of pressure rapidly and uniformly, independently from the size and geometry of the product [17,18]. High pressure is known to affect the conformation of macromolecules by disrupting hydrophobic and electrostatic interactions, the transition temperature of lipids and water, and the kinetics of many chemical reactions, while not affecting covalent bonds. Phenomena that are accompanied by a decrease in volume are enhanced by pressure, and vice-versa (principle of Le Chatelier); therefore, under pressure, reaction equilibriums are shifted towards the most compact state [17,18,19,20,21,22]. The use of HHP can modify food properties and induce denaturation of proteins by shifting the delicate equilibrium between interactions that stabilize the folded conformation of native proteins. Such effects may also impact protein behavior in solution and gelation. HHP may also modify protein’s functional properties by the formation of new bonds [23,24]. The first report of protein gelation under pressure was already in 1914 [25]. Proteins, such as egg proteins, actomyosin [19], soy proteins [19,26], whey proteins [27,28], sweet potato protein [29], and others, were shown to gel under HPP conditions. In pressure-induced gelation, high pressure changes the conformation of proteins leading to denaturation or aggregation as a function of the protein system and the used conditions. A few previous studies comparing thermal and pressure-induced gels reported that pressure-induced gels are usually softer or weaker compared to heat-induced, but resistant to breaking [13,27,30], likely originating from a different gelation mechanism [19].

Potato proteins can also form gel networks [31], with the major protein in potato, patatin, which has a lower thermal denaturation temperature compared to other well-known proteins (β-lactoglobulin, ovalbumin and glycinin) [32,33]. However, the type of protein extraction and the extracted protein groups might have a major effect on gelation, as others reported that the gelation of potato protein is poor [34]. Thermal gelation of potato protein was reported to result in the weakest gel at pH ~5 and stronger gels below and above this pH [33,35]. Information regarding HP induced gelation of plant-based proteins is limited in general, and especially regarding the gelation of fruit and vegetable proteins [36]. Aggregation of fully solubilized potato protein at low concentrations by HHP was previously reported [37] and the formation of a new band on an SDS-PAGE was reported for purified patatin treated by high pressure (250–550 MPa), suggesting the possibility of protein-protein interactions as a result of pressure application [24].

Our literature review suggests significant knowledge gaps regarding thermal and pressure gelation of potato proteins. No information is present regarding pressure-induced gelation of potato proteins and minimal information exists regarding properties of heat-induced gels from this alternative plant-based protein source. The current work aimed to study and compare the possibility and properties of thermally and HHP formed gels from commercial potato protein isolate at two pH levels modeling acidic and neutral conditions.

## 2. Materials and Methods

### 2.1. Materials

Commercial potato protein isolate (PPI), (Solanic^®^ 200, batch no. 410000000) was obtained from Avebe, Veendam, Netherlands. Protein purity was 90.5%, fiber was 3.5%, while carbohydrates were below 0.2%, fat was 0.2%, and salt was 0.04% as stated by the manufacturer. Na_2_HPO_4_ and NaH_2_PO_4_ were purchased from Spectrum Chemical, New Brunswick, NJ, USA. o-phosphoric acid 85%, acetic acid glacial and coomassie brilliant blue r-250 were purchased from Bio-lab, Ashkelon, Israel. Ethanol 96% was purchased from Gadot, Haifa, Israel. Castor oil pharma grade, silicon oil, water HPLC, acrylamide 40%, ammonium persulfate, Sodium dodecyl sulfate (SDS), trizma base, glycine, DL-dithiothreitol 99% (DTT), and bromophenol blue were purchased from Sigma Aldrich, St. Louis, MO, USA. L-(+)-ascorbic acid 99% and *N,N,N′,N′*-Tetramethylethylenediamine were purchased from Alfa Aesar, Haverhill, MA, USA. Meta-phosphoric acid, Formic acid 89–100% were purchased from Merck Millipore, Burlington, MA, USA. Acetonitrile was purchased from J.T.Baker, Warren County, NJ, USA. PageRuler plus prestained protein ladder (#26619) was purchased from Thermo Scientific, Vilnius, Lithuania.

### 2.2. Methods

#### 2.2.1. Aggregation/Gelation by Pressure or Heat

**Figure 1:**
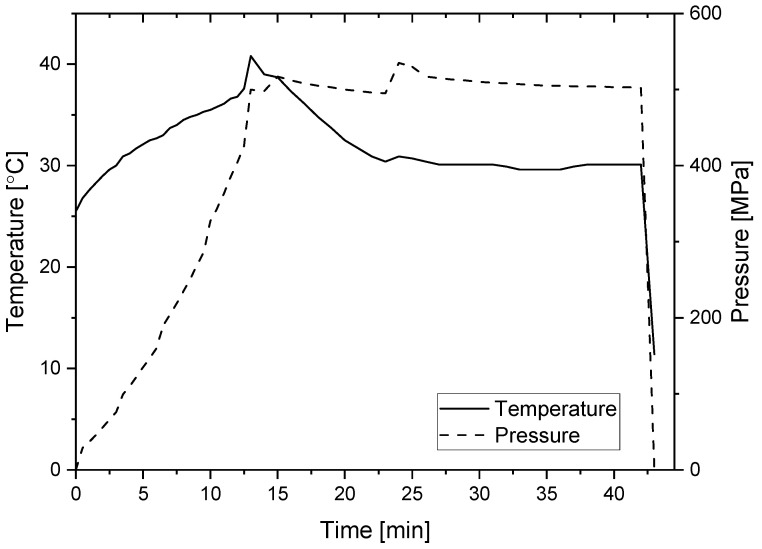
Representative time-temperature-pressure profile of the process used for the gelation of potato protein isolate at 500 MPa (temperature was measured at the pressurizing liquid).

In total, 10% or 1% (*w*/*w*) of PPI was dispersed in phosphate buffer in two pH levels pH = 3 and pH = 7 (20 mM) and stirred for 30 min. pH conditions were selected to represent high and low acid foods while being similarly far from the pI of the main protein of PPI—patatin (~5). PPI solutions with a suitable volume (500 µL or 5 mL according to the experiments) were vacuum-sealed (Henkelman, Ashford, Kent, UK) in two layers of polyethylene bag. Pressure was applied using the S-FL-850-09-W system (Stansted Fluid Power Ltd., Harlow, Essex, UK), while thermal gelation was obtained using a temperature-controlled water bath. For the thermally formed gels, five different temperatures (45 °C, 50 °C, 55 °C, 60 °C, and 90 °C) were tested. Hydrostatic pressure was applied at 300 MPa, 400 MPa, and 500 MPa. As the time required for gelation and also gelation kinetics were not examined in the current project, samples were treated for 30 min based on previously reported studies [19,38], thus allowing sufficient time for the heat transfer in the thermally formed gels. Temperature profile during pressure experiments (temperature increase due to adiabatic heating) was determined in the pressurizing liquid and not the sample itself, and the temperature was restricted not to pass 40 °C. The temperature restriction was applied to prevent thermal gelation of the pressure formed gels as our preliminary experiments presented the start of thermal gelation of this protein at ~45 °C. Important to note is that in industrial units operating with water as a pressure transmitting fluid such restriction will not be needed as long as the starting temperature is 25 °C or below as the adiabatic heating in water is lower than in the EtOH-castor oil (80:20) used in our system as the pressure transmitting fluid. A representative pressure-temperature profile showing the temperature increase in the vessel (the pressure-transmitting fluid) due to the adiabatic heating when 500 MPa was reached can be seen in Figure 1. During pressurization, the pressure treatment cell was cooled using FP51-SL Ultra-Low Refrigerated-Heating Circulator (JULABO GmbH, Seelbach, Germany). Before each pressure treatment, the cell was equilibrated to a temperature that at the end of the pressurization build up step will always reach 39–40 °C, to make sure that temperature variation between pressure levels due to the adiabatic heat is not the major reason for the difference in properties between the different pressure levels. The pressure ramp was ~40 MPa/min.

#### 2.2.2. Molecular Weight Determination Using Sodium Dodecyl Sulfate Polyacrylamide Gel Electrophoresis (SDS-PAGE)

The molecular weight (Mw) profile of the heat and pressure treated PPI and the molecular weight of the protein in the solution released after water holding capacity assay (2.2.6) was determined by sodium dodecyl sulfate polyacrylamide gel electrophoresis (SDS-PAGE). All protein samples were diluted with sample buffer (50% glycerol, 10% SDS, 0.5 M tris, 10 mM EDTA, bromophenol blue, and 13% water) and DL-dithiothreitol (DTT) (0.5 M) was added; then, the samples were heated for 10 min at 70 °C and centrifuged before loading on the gel (10% acrylamide). A molecular weight marker (10 kDa–250 kDa) was used to estimate the molecular weight of the proteins. The running conditions were based on a previous publication [39]. To estimate the percentage of patatin protein from the total detected protein, gel densitometry was performed using ImageJ software [40]. Image brightness of the scanned SDS-PAGE was adjusted using GIMP version 2.10.10.

#### 2.2.3. In Situ Spectroscopic Study of the Effect of Pressure on Potato Protein Isolate

PPI solutions (0.05% *w*/*v*) were prepared in the two studied buffers (20 mM phosphate buffer pH 3 and 7) daily. The samples were vortexed and 3.8 mL was transferred to the high-pressure optical cell. The intensity of the transmitted light through the studied solutions at the varying conditions was measured in situ in a thermostat (Hakke DC30/K10 circulating chiller, Thermo Scientific, Waltham, MA, USA) high-pressure optical cell (Type 740.2330 from SITEC Sieber Engineering AG, Maur, Switzerland) that is equipped with a manual pressure generating system (750.1700), optical cell, and a separation piston as previously described [41]. Fiber optic cables connect the optical cell to a UV-VIS spectrometer (Evolution 260, Thermo Scientific, USA). To evaluate the effect of pressure and heat on PPI, the absorbance of the samples between 240–300 nm is presented at room temperature and at 40 °C as a function of pressure (300–500 MPa) and at atmospheric pressure as a function of temperature (25 °C, 40–85 °C) at two points of time: immediately and after 30 or 120 min of incubation. Appropriate buffer controls were subtracted from the relevant samples to obtain the absorbance of the protein only, 9 pt Savitzky-Gilay smoothing was applied to reduce noise. The experiment was performed in duplicate and the spectrum is reported as the average value.

#### 2.2.4. Rheological Properties

The rheological properties of the gels were determined using small deformation analysis to probe their viscoelastic properties within the linear viscoelastic region (LVR) [42,43] using Discovery HR-2 (TA Instruments, New Castle, DE, USA). To determine the correct parameters, the LVR was obtained using oscillation strain sweep at a frequency of 1 Hz or 10 Hz and a strain range of 0.01–100%. The storage modulus (G′) and loss modulus (G″) were recorded as a function of frequency.

##### Frequency Sweep

After pressure or thermal gelation, 10% *w*/*w* protein gel samples were transferred from the vacuum-sealed bags and studied at least 1 h and no more than 3 h after treatment. Gels were placed on the rheometer lower base plate and then cut to an 8 mm radius; the upper 8 mm parallel plate probe was lowered to a 1.5 mm gap. The storage and loss modulus were recorded as a function of frequency at 25 °C at a constant strain of 1%, which was within the LVR and frequency range, between 0.05–100 Hz [44,45]. The rheological measurements were conducted in triplicate.

The storage modulus as a function of frequency can provide information on gel characteristics [43]. The dependence of the storage modulus with frequency was fitted using Equation (1) [46]:

G′ = K(ω)^n^ or log G′ = n log ω + K (1)

where G′ is the storage modulus, ω is the oscillation frequency, K is a constant, and the constant n is the slope in a log-log plot. The obtained the fit parameters n and k were used to compare gel characteristics.

#### 2.2.5. Texture Profile Analysis (TPA)

Mechanical performance analysis was conducted using a two-cycle penetration test by texture analyzer TA1 (LLOYD, Bognor Regis, West Sussex, UK). Samples for the TPA analysis were prepared by placing 10% (*w*/*w*) potato protein solution in a Teflon bottle (5 mL, purchased from Simada, Holon, Israel), which was vacuum-sealed in a polyethylene bag and treated using thermal or pressure conditions. At least 1 h after pressure or heat treatment, the samples were analyzed according to a previously reported method [46]. The samples were penetrated twice to 50% of their original height using a 10 N load cell and a crosshead speed of 0.3 mm/s using 5 s waiting time with a 4 mm probe at room temperature. Gels were analyzed using NEXYGENPlus 3.0 data analysis software to compare the textural properties of obtained gels. The following parameters were used for comparison based on previous studies: hardness (peak force in the first compression cycle), adhesiveness (the negative work generated during the upstroke of the probe), and cohesiveness (ratio of the positive area during the second compression cycle to that of the first compression cycle) [46,47]. At least six replicates were tested for each sample.

#### 2.2.6. Water Holding Capacity (WHC)

In total, 500 µL of protein solution at pH 3 was vacuum-sealed followed by heat or pressure treatment for 30 min to prepare a gel. After at least 1 h, the WHC of the gels was determined according to previously-reported methods [48,49,50]. A centrifuge filter unit composed of nylon filter (0.45 µm) and a 2 mL micro-centrifugal tube (Micro-Centrifugal Filters (F2517-4), Thermo Scientific, USA) was used. All samples were carefully transferred and placed on the filter in the micro-centrifugal tubes. Centrifugation was performed at 5000 g for 30 min at 24 °C, and Equation (2) was used to determine WHC (%):

(2) $$\%\mathrm{WHC}=\frac{{\mathrm{filter}}_{2}-{\mathrm{filter}}_{1}}{\mathrm{gel}}\times 100$$

where filter_2_ is the weight of the filter after centrifuge (mg), filter_1_ is the weight of the empty filter (mg), and gel is the weight of the gel before centrifuge. Five replicates were examined for each sample. The buffer containing protein that was released from the gels during WHC study and passed the filter was also studied using SDS-PAGE as described in Section 2.2.2. Buffer released from the pressure-induced gel was diluted by 20, from heat-induced gels (except for 90 °C) buffer was diluted by 7, from heat-induced gels formed at 90 °C buffer was diluted by 2. The untreated potato solution after the same experimental set-up as the gels was diluted by 40, and the PPI sample was directly loaded (14 µg of protein). The WHC study was conducted in triplicate.

#### 2.2.7. Vitamin C Determination—Comparison of Degradation during Gelation of a Thermally Liable Compound

In order to compare the effect of thermal and high-pressure gelation in terms of possible preservation of thermally liable compounds, gelation was performed in a system enriched with vitamin C as a model compound. 10% *w*/*w* potato protein in phosphate buffer was mixed with 1 mg/mL ascorbic acid. The examined system (5 mL) of the potato protein with ascorbic acid was vacuum-sealed and then treated with heat or pressure for 30 min to form the vitamin C enriched thermal and high-pressure assisted gels. After pressure or heat treatment, Vitamin C was extracted and quantified. Specifically, samples were homogenized (OMNI International Inc., Waterbury, CT, USA) with 10 mL 5% meta-phosphoric acid for 1 min. The homogenate filtered (Whatman paper 110 mm) and the residue was treated with 5 mL of 5% meta-phosphoric acid for two additional extractions. The filtrate was combined and centrifuged at 4000× *g* for 10 min at 10 °C. The supernatant was collected and made up to 25 mL and then filtered (0.45 µm) [51]. To quantify dehydroascorbic acid (DHA), 0.2 mL of DTT (20 mg/mL) solution was added to a 1 mL sample and stored in dark conditions for 2 h before analyses (0.45 µm) [52,53,54]. The samples (with and without DTT) were then injected to HPLC 1260 infinity (Agilent, Santa Clara, CA, USA) equipped with Poroshell 120 EC-C18 column (2.1 × 150 mm, 1.9 µm particle size). Analysis of vitamin C was carried out using a varying flow-rate with a mobile phase of water with formic acid (0.1%, *v*/*v*) and acetonitrile; at 23 °C, the detection was performed by UV-DAD detection (245 nm). Injection volume was 5 µL and total run time was 13 min. Elution profile: at 0–3 min the flow changed from 0.19 to 0.17 mL/min and from 3–6 min the flow increased to 0.23 mL/min and the mobile phase changed from water with 0.1% formic acid to acetonitrile. From 6–8 min, the flow was 0.23 mL/min. From 8–10 min the mobile phase changed from acetonitrile to water with formic acid and from 10–13 min (flow 0.23 mL/min) remained at water with formic acid. The DHA content was calculated as the difference between vitamin C after reduction (DHA + AA) and vitamin C (AA) without reduction. Six ascorbic acid standard samples were used for a calibration standard curve (30–250 µg/mL ascorbic acid in meta-phosphoric acid). The test was performed in duplicate.

#### 2.2.8. Statistical Analysis

One-way ANOVA with a post-hoc Tukey (Honestly Significant Difference) test was performed for the determination of statistically significant differences. The number of repetitions is reported for each experiment separately in the materials and methods.

## 3. Results and Discussion

### 3.1. Effect of Heat or Pressure on the Molecular Weight of Potato Protein Isolate Solution

**Figure 2:**
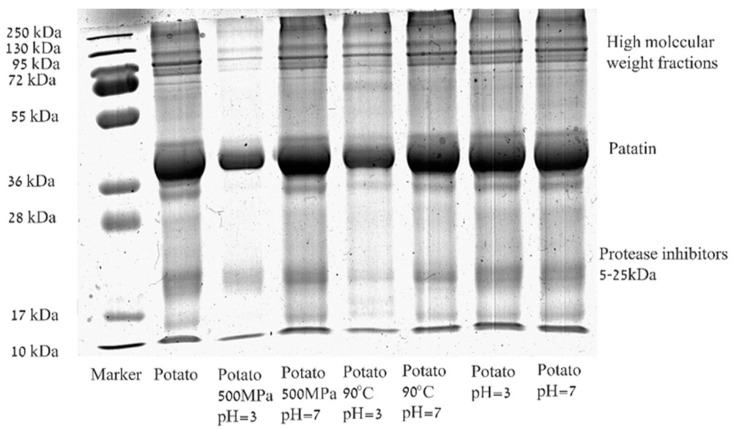
Representative Sodium Dodecyl Sulfate Polyacrylamide Gel Electrophoresis (SDS-PAGE) of potato protein isolate solution (1% *w*/*w*) at two pH levels (7 and 3) before and after thermal (90 °C) and pressure (500 MPa) treatments for 30 min.

The SDS-Page of potato protein isolate (PPI) solution (1% *w*/*w*) at the two studied pH levels (7 and 3) before and after heat and pressure treatments is presented in Figure 2. The pH levels were selected to represent a model for neutral and acidic products and to be sufficiently and similarly far from the isoelectric point of the major protein in PPI–Patatin pH 4.9–5.25 [5,11]. It can be seen that the untreated commercial PPI solution contains proteins with varying molecular weights including proteins that are smaller than 10 kDa (based on the band appearance at the bottom of the gel). In addition, some large aggregates/insoluble proteins, larger than 250 kDa, were noted on the top of the stacking gel (not shown). The molecular weights present in untreated PPI solution are similar to previously published results for potato proteins [23,34,55], showing the three groups of proteins: patatin, protease inhibitors, and high-molecular-weight proteins. According to our results, the used PPI in this study consists of ~60% patatin (Mw 45–50 kDa), based on gel densitometry performed by ImageJ software, which is higher than the previously reported 40% [5]. Important to note that not all the proteins were observed clearly, or had a very thin band that did not contribute to the quantification with ImageJ, and some proteins did not enter into the gel (not shown); therefore, this value represents only an estimation of the relative protein fraction capable of entering the SDS gel.

While the SDS-PAGE profiles of PPI in the buffer solution at pH 3 and 7 do not differ from each other, a difference is observed after thermal and pressure treatments. At pH = 3 a decrease in intensities of all bands in the protein pattern is noticed, compared to the unprocessed PPI solution. It should be noted that after both heat and pressure treatments at pH = 3, aggregation is visually observed, and only the soluble proteins are analyzed by SDS-PAGE. Therefore, the observed lower quantity of protein is likely to be the result of aggregation. It was reported that potato proteins unfold between 55 °C and 75 °C with a stabilizing effect for ionic strength [10]. Patatin (1 mg/mL) at pH 7 (without added NaCl) was previously reported to fully solubilize without an effect for thermal treatment, while the protease inhibitors fraction was shown to undergo thermal coagulation [56]. Others suggested thermal coagulation of patatin (0.33 mg/mL) at temperatures above 50 °C with the SDS-PAGE in non-reducing (native) conditions showing three bands, while the addition of β-mercaptoethanol resulted only in the presence of monomeric patatin, indicative of the formation of sulfur bridges in the native protein. However, it was also suggested that the formation of sulfur bridges is not the determining mechanism of the aggregation [57]. Pure patatin (2% *w*/*w*) was reported to aggregate at pH = 7 after pressure treatment (15 min at 250 MPa, 350 MPa, 450 MPa, and 550 MPa) resulting in a band with a molecular weight of 130 kDa (in reducing SDS conditions) that was shown to increase as the pressure increased [24]. Baier and Knorr [37] reported conservation of 93% of the protein solubility after isothermal treatment at 20 °C and 400 MPa (pH 7), while isothermal treatment at 40 °C and 600 MPa resulted in the aggregation of ~30%. Others also suggested pressure-induced aggregation of myofibrillar proteins due to the intermolecular disulfide bond formation [58]. Such a result characterized by the formation of a new band with a higher molecular weight was not observed in our case, likely due to the reduced sample preparation conditions we used for SDS analysis. An alternative explanation could be the overlapping proteins observed in the region of 130 kDa, or because the presence of other proteins (in addition to patatin) in the isolate significantly changed the aggregation profile. Pressure elevation will result in a reduced volume of the protein in the solution by reactions or interactions promoting a lower total volume (le Chatelier principle) [59]. However, the impact of pressure on proteins behavior cannot currently be derived from known protein structural features, and therefore, often requires separate testing [37].

### 3.2. In Situ Spectroscopic Study of the Effect of Pressure on PPI in Solution

**Figure 3:**
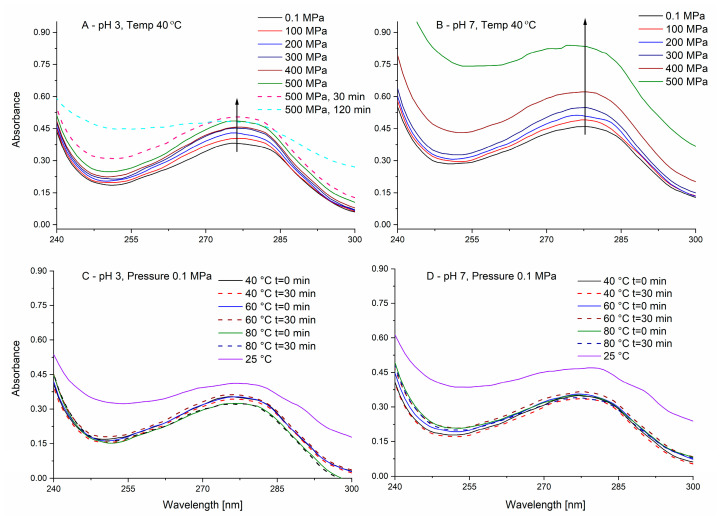
Averaged UV absorbance of potato protein isolate (PPI) (0.05% *w*/*w*) during pressure (**A**,**B**) and thermal (**C**,**D**) treatments at pH 3 (**A**,**C**) and pH 7 (**B**,**D**). Full lines represent the absorbance immediately after pressure or temperature increase, while the dashed lines represent the absorbance after different times as described in the legend. The arrows are to guide the eye for changes occurring with increasing pressure.

As in protein gelation, under pressure or heat, the denaturation step is of major importance, a better understanding of the effects of pressure on the denaturation is of interest. However, such studies are hard to perform in the common HHP equipment because upon pressure release, protein structure may change. Spectroscopic methods in optical cells allowing in situ measurement during the pressurization step itself can be of assistance, yet they require less prevalent equipment. Spectroscopic analysis (usually in the UV or the IR regions) during the pressurization allows studying the effects of pressure and not just the outcome on the final product. The UV region of proteins is dominated by the absorbance of phenylalanine, tyrosine, and/or tryptophan [60]. As PPI is not a purified single protein, a complete understanding of the effects of protein in such system using derivative spectroscopy, which was reported as a tool for analyzing the effects of pressure on proteins conformation [61], is not feasible (different proteins have different amino acids and different denaturation parameters resulting in a superposition of effects). On the other hand, a general comparative view of the effects of pressure and temperature can be obtained. In the current work, we have studied the effects of pressure on PPI while keeping the maximal temperature achieved during pressurization in the gelation experiments (40 °C) as constant (as an upper threshold for the effect as was used in the gelation studies). Spectral studies are performed at much lower concentrations compared to gelation as treatments in proteins concentration of less than 0.1% were suggested to minimize possible aggregation phenomena [62], therefore, allowing to focus on denaturation. For PPI solutions (0.05% *w*/*w*, Figure 3), a typical protein UV spectrum is observed with an absorbance maximum at ~278 nm. As can be seen in Figure 3A, in pH 3 a small increase in the absorbance can be noticed immediately with increasing pressure while maintaining the maximal pressure (500 MPa) for 30 and 120 min resulted in a further spectral increase, yet mostly not around the peak maximum. It is likely that the immediate pressure-dependent increase originates from small changes in the path length and/or the effective concentration of the protein (water is compressed by ~14–15% at 500 MPa), although such phenomenon was also previously explained by the exposure of aromatic amino acid residues [63,64]. The observed change in absorbance at pH 3 with time is possibly related to pressure-induced denaturation, although protein-protein interactions resulting in some initial aggregation cannot be excluded, despite the very low concentration used. On the other hand, application of pressure at pH 7 (Figure 3B) resulted in a much larger and immediate absorbance increase, especially for 400 and 500 MPa. Recording the absorbance spectrum with time at the maximal pressure reached (500 MPa) resulted in saturation of the absorbance signal (>2.5) and is not presented. Such an extensive increase in absorbance can be, at least partially, attributed to enhanced protein-protein interactions due to pressure-induced pH shifts. The pH of phosphate buffer is known to decrease at elevated pressures [41,65] and when the solution is at pH 7 (before pressurization), elevated pressures would bring the system closer to the pI of patatin, therefore, reducing the solubility [15]. Heating at the two pH levels (pH 3 and 7, Figure 3C,D) did not result in significant spectral changes even when higher temperatures than 40 °C were tested, despite the reported unfolding and aggregation of PPI proteins above 55 °C [10,37]. A decrease in the absorbance was observed due to heating from 25 °C to 40 °C. However, such decrease is likely related do reduced light scattering and not absorbance due to residual aggregates present at 25 °C, as samples at 25 °C presented residual absorbance also at 600 nm, which did not appear at samples to 40 °C nor at high-pressure samples (at 40 °C, not shown). The decrease in turbidity is likely related to increased solubility of some potato proteins by heating below temperatures resulting in the destabilization of secondary and tertiary structures [66]. We did not observe a clear pressure-induced blue shift of the UV spectrum that was suggested to occur due to hydration of hydrophobic residues upon exposure to the solvent, nor did we see a reported red shift that can happen due to a change in the environment of Tryptophan [67]. The lacking detectible peak shift by heat/pressure, despite the reported denaturation of patatin, possibly originates from the multiple existing proteins in the isolate with different pressure/temperature stabilities and the utilization of a smoothing algorithm needed for the in situ HPP-spectroscopic system due to a larger background noise compared to regular spectroscopy.

### 3.3. Dynamic Oscillatory Rheological Properties of Thermally and Pressure-Induced Gels

**Figure 4:**
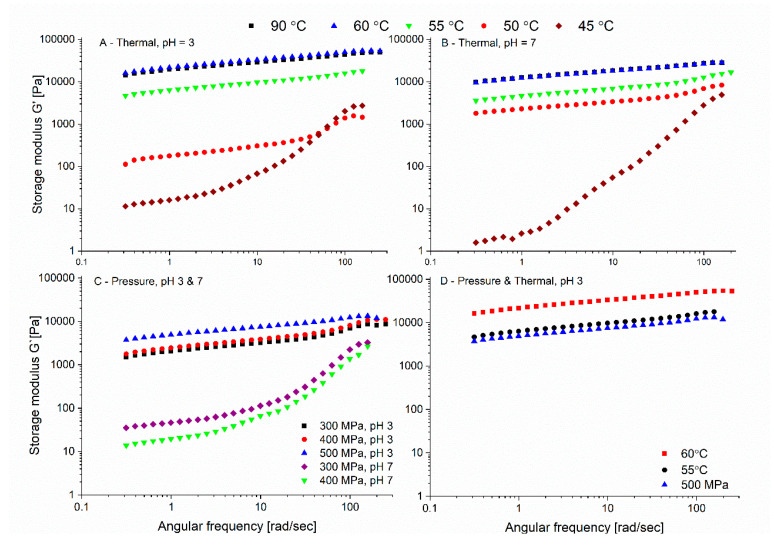
Storage modulus as a function of the angular frequency of potato protein isolate gels (10% *w*/*w*) at pH = 3 and pH = 7. (**A**,**B**) thermal gelation at 90 °C, 60 °C, 55 °C, 50 °C, and 45 °C at pH = 3 and pH = 7, respectively. (**C**) Pressure-induced gelation at maximal pressures of 300, 400, and 500 MPa, buffer initial pH 3 and 7. (**D**) Comparison between the pressure-induced gel formed at pH 3 at 500 MPa to thermally formed gels (60 °C and 55 °C).

The rheological properties of 10% *w*/*w* thermal and pressure induced gels at different pH levels were characterized by frequency-dependent dynamic oscillatory measurement, see Figure 4. The storage modulus represents the elastic component of the network and is a measure for the strength of the structure contributing to a three-dimensional network, while the loss modulus is a measure of the viscous component. For both thermal and pressure gels, the storage modulus was higher than the loss modulus over the whole frequency range, indicating that the elastic contribution was more than the viscous contribution; thus, a gel state can be determined (data not shown) [68]. The parameters (n, K) obtained from by fitting the storage modulus as a function of frequency using Equation (1) can provide valuable information regarding the gel structure and characteristics. Such analysis can identify the difference between entanglement network, covalently cross-linked materials, and physical gels [42,69]. Generally, food gels exhibit slightly frequency-dependent behavior, which implies that they have a physical nature [70]. The n value expresses the nature of the gel network, with *n* = 0 implying a cross-linked gel and n > 0 suggesting a physical gel. Therefore, n can be used as a measure of the proximity of the gel characteristics compared to covalently cross-linked gel [9,46,69]. The values of K reflect the gels’ storage modulus value at the minimum frequency, i.e., intercept of the storage modulus with y axis, which can present the levels of molecular interaction in the gel matrix. A higher K value is suggestive of a stronger molecular interactions [9]. The fitted n and K parameters based on Equation (1) are presented in Table 1. An absence of fitted parameters in the table suggests that either no gel was formed or that a fit to Equation (1) could not be applied.

Most thermally formed gels exhibited a good fit for Equation (1), thus confirming that PPI gels are physical gels. At pH 7 and 45 °C the fit was applied starting from 1 Hz frequency due to fitting limitation at lower frequencies; therefore, the value will be treated with caution.

Previous results reported the formation of patatin gels at pH 7 from 50–55 °C (depending on ionic strength) [32], in accordance with the observed formation of physical gels from PPI at 45–50 °C in this study. Also important to note is that we only tested the rheological properties after 30 min, and while less often reported, gelation kinetics is also of importance, and much longer heating times can possibly result in gelation even at lower temperatures [15]. As we aimed to compare pressure-formed gels thermally, and no equipment allowing pressure-induced gelation in the rheometer is available, both thermal and pressure gels were formed outside of the rheometer. Our results reveal an increase in the storage modulus with increasing temperature below 60 °C over the whole frequency range (Figure 3A,B), without further increase when the external temperature used for gelation increased to 90 °C. Similar to our results, temperature sweep studies reported that after the gelation point, there is a further increase in the storage modulus until reaching a plateau [71,72,73,74,75].

When comparing the slopes of the mechanical spectrum, which can be a measure of gel similarity to a covalently bonded gel, at the same applied temperature for the two pH levels, only the slope at 50 °C was statistically different (Table 1). While the constant K was also different when the gels were formed at 55 °C and 60 °C.

The trend, as presented in Table 1, showing a decrease of the slope (n) with increasing temperature, combined with an increase in K (especially in the lower gelation temperatures), suggests a stronger gel network that behaves closer to a chemically cross-linked network [9]. The results show that as the temperature increases above the gelation temperature there is an increase in the storage modulus (but only up to a certain temperature), which is indicative of a more solid behavior and stronger gels.

For the pressure-induced gels, the maximum temperature reached in the pressure cell (for a short time as seen in Figure 1) during the pressure treatment due to the adiabatic heating was lower (just below 40 °C) than the minimum temperature showing gelation by heat both in our preliminary studies (not shown) and in the literature. Patatin, the major protein in potato protein isolate was reported to be stable up to 50 °C [57] with the tertiary structure being stable up to 45 °C [5], although others suggested tertiary structure changes of patatin starting from 28 °C [76]. At all pressures, the maximum temperature reached at the end of the pressure build-up was identical, achieved by slightly changing the starting temperature, to make sure that the temperature is not the major reason for the difference in gel formation (between the pressure formed gels).

As could be observed visually (not shown), and presented in Figure 4C, at pH 7, pressures of 300 and 400 MPa resulted in the formation of fragile gels, characterized with low storage modulus, which was still higher than the loss modulus across the frequency range but could not be fitted to Equation (1). The rheological curves of those samples were similar to the curve of the thermal gels formed at 45 °C at pH 3. Pressurization at 500 MPa at pH 7 did not result in any gel formation. On the other hand, at pH 3, with the studied pressure levels, physical gels that could be fitted to Equation (1) were formed. The rheological profile (Figure 4D) of the pressure-induced gel at pH 3 (300 MPa–500 MPa) was very similar to the profile of the heat-induced gel at 55 °C but lower than that of the thermal gel formed at 60 °C. As pressure increased, we observed a mild and not statistically significant trend for n decrease and a significant increase in K between 500 MPa compared to 300 MPa and 400 MPa (Table 1).

Important to note is that when the maximal temperature in the pressure transmitting fluid did not surpass 30 °C (by starting the pressurization at even lower temperatures, ~10 °C and ~20 °C, with the same protein concentration), no stable gels were formed using pressure treatment, including at pH 3 (after 30 min), suggesting a possible contribution of the temperature to the pressure gelation. It is not fully clear whether the required thermal contribution is crucial for the denaturation or the aggregation steps. Furthermore, we did not test if longer pressurization times may allow the formation of gels by pressures even at lower maximal temperatures. However, the observation can be related to the previously reported weakening of the tertiary structure of patatin already at 28 °C [76] or to a contribution to the aggregation step as the temperature can affect hydrophobic interactions, hydrogen bonds, and covalent disulphide bonds. It has been published that increasing the temperature (at the same pressure) increases the hardness of ovalbumin and soy protein gels [77], confirming that heat can contribute to the formation of a more rigid gel structure for pressure-induced gels.

The reason for the lack of formation of physical gels by pressure at pH 7, despite the clear spectral change of PPI solution in this pH observed in Figure 3, needs future attention. We suggested that it is related to pressure-induced pH shifts of the buffer, bringing the solution closer to the pI of patatin and reducing solubility, which is important to form macroscopically homogeneous hydrogels [15]. As it is well known that changes in pressure can alter the K_a_ of weak acids and bases, resulting in significant pH shifts in numerous buffers, but also in real foods [78] and even in protein solutions (without buffers) [79,80], the point of the impact of pH shifts on pressure induced gelation deserves specific focus in future studies.

### 3.4. Comparison of the Instrumental Texture of the Pressure and Thermally-Induced Physical Gels at pH 3

As physical gels were formed by pressure and heat only at pH 3, further characterization focused only on this pH. Texture profile analysis (TPA) was used for evaluation of gel texture. Such measurements are useful for routine analysis of food gel texture [47].

**Figure 5:**
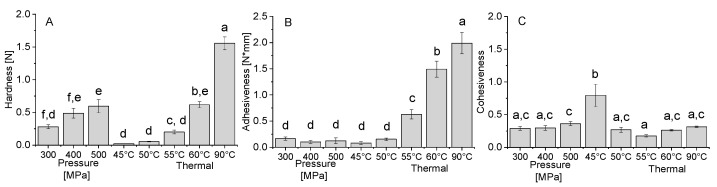
Texture profile analysis data of hardness (**A**), adhesiveness (**B**), and cohesiveness (**C**) of the heat and pressure induced gels at pH 3. Error bars represent standard deviation, different letters indicate significant differences (*p* < 0.05, *n* = 6).

The results in Figure 5A–C report the obtained hardness, adhesiveness, and cohesiveness obtained by TPA of the gels formed at pH 3 by heat and pressure.

**Figure 6:**
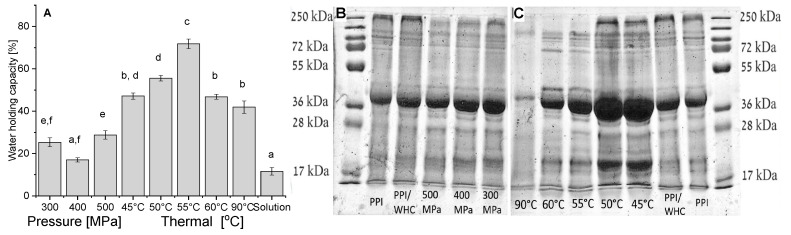
Water holding capacity (**A**) of the heat and pressure induced gels at pH 3. SDS-PAGE of potato proteins that were released from pressure (**B**) and thermal (**C**) induced gels (pH 3) during the Water Holding Capacity (WHC) experiment. The buffer released from the pressure-induced gel was diluted by 20, from thermally-induced gels, except for 90 °C, the buffer was diluted by 7, from thermally-induced gels formed at 90 °C the buffer was diluted by 2, the untreated potato solution after the same experimental set-up as the gels (PPI/WHC) was diluted by 40, and 14 µg PPI was loaded. Different letters (in **A**) indicate significant differences (*p* < 0.05, *n* = 5), error bars indicate standard deviation.

The results reveal that the TPA hardness of the heat-induced gels significantly increased with increasing temperature. The increase in pressure led to a more moderate effect on the hardness, though a significant increase was observed between 300 MPa and 500 MPa. The pressure-induced gels at 500 MPa show a similar hardness to the heat-induced gels at 60 °C. The adhesiveness of the gel, which is related to the forces needed to overcome the attractive forces between the food and other materials with which it comes in contact [47], increased with increasing temperature (Figure 6B). On the other hand, no effect was observed for the increase in pressure, and in general, the adhesiveness of pressure formed gel was lower than of thermally formed gels. A previous study comparing the hardness and adhesiveness of thermal and pressure-formed soy gels [30] reported a similar result showing higher hardness and adhesiveness (absolute value) for thermal gels compared to pressure-induced gels of soy protein isolate, 7S and 11S soy fractions. Similarly, egg white proteins presented higher hardness and adhesiveness for thermal gels compared to pressure-induced gels (Okamoto et al., 1990). While we also observed an increase in hardness with increasing pressure, we did not observe a decrease in adhesiveness, possibly due to the very low adhesiveness of high-pressure gels in general, or the specific properties of the PPI. The cohesiveness is indicative of the strength of internal bonds of food body and the degree to which it can be deformed before it ruptures (breaks) [81]. It can be seen in Figure 5C that almost all the gels have similar cohesiveness values, except the thermally formed gel at 45 °C, which has a higher value, while it was the gel with the lowest storage modulus (Figure 4A). Similar cohesiveness behavior was also seen in soy protein gels while comparing pressure-induced gels and thermally-induced gels [30]. The difference between the textural attributes of thermal and pressure-assisted gels, is indicative, in addition to the expected difference in the sensorial properties, of differences in gel microstructure.

### 3.5. Comparison of the Water Holding Capacity (WHC) and the Involvement of Various PPI Proteins in Gelation of Thermal and Pressure Physical Gels

The WHC is a physical property, defined as the ability of a matrix to hold its own and added water during the application of forces (like centrifugation) [82]. It can be seen from Figure 6A that the WHC of heat-induced gels has a maximum at 55 °C. It was reported that for thermal gelation of globular proteins the finest structure is received under conditions during which the tendency for aggregation is just above the needed temperature for the formation of gels. While increased random aggregation above such temperature will result in a coarser structure, related to poorer water-holding properties [83]. Pressure-induced gels have lower WHC than heat-induced gels in general, and with no significant differences at the different pressures except between 500 MPa and 400 MPa. A similar outcome showing lower WHC of pressure compared to thermal gels was also previously reported for gels from Blue Whiting (*Micromesistius poutassou*) muscle proteins [84]. Protein solution that was not subjected to pressure or heat treatment has a significantly lower WHC, most of the water was released but some did not pass the centrifugation filter.

To gain some additional insights into the mechanism of PPI gelation, due to the broad range of present proteins in the isolate, we have studied, using SDS-PAGE, which proteins were released during the WHC study (therefore suggested to be less involved in gel structure). Such knowledge can provide additional information regarding the proteins involved in the gel formation as opposed to the ones that were released. Due to a different concentration of protein in the released buffer, samples were diluted differently, and therefore, only relative intensities of the bands in the same line should be considered. It can be seen from Figure 6B,C that a wide range of various proteins were released in most of the gelation conditions, with no new bands appearing compared to PPI solution.

Our results reveal that the higher the temperature (Figure 6C), the larger the involvement of the proteins in the gel formation. This observation is well-correlated with the hardness (TPA) increase with increasing temperature (Figure 5A). The larger proteins with molecular weights of ~250 kDa seem to be the most involved in the gel formation as their band completely disappeared at gelation temperatures above 55 °C and also continuously decreased with increasing pressure (Figure 6B, all samples from pressure-induced gels were diluted identically). Patatin is visible in all lanes, suggesting that it is not fully involved in the protein network formed. Such an outcome can be supported by the fact that purified patatin remained practically fully soluble at 40 °C and 600 MPa (pH 7), while at the same conditions the solubility of potato protein concentrate decreased by ~30% [10]. It was also previously suggested that only a small part of potato proteins became insoluble above 60 °C at low ionic strength [10] possibly indicative of some stability of this specific protein in the PPI (or different sub-groups) during thermal treatment.

### 3.6. Stability of Vitamin C as a Model Thermally Liable Compound During Thermal and Pressure Gelation

**Figure 7:**
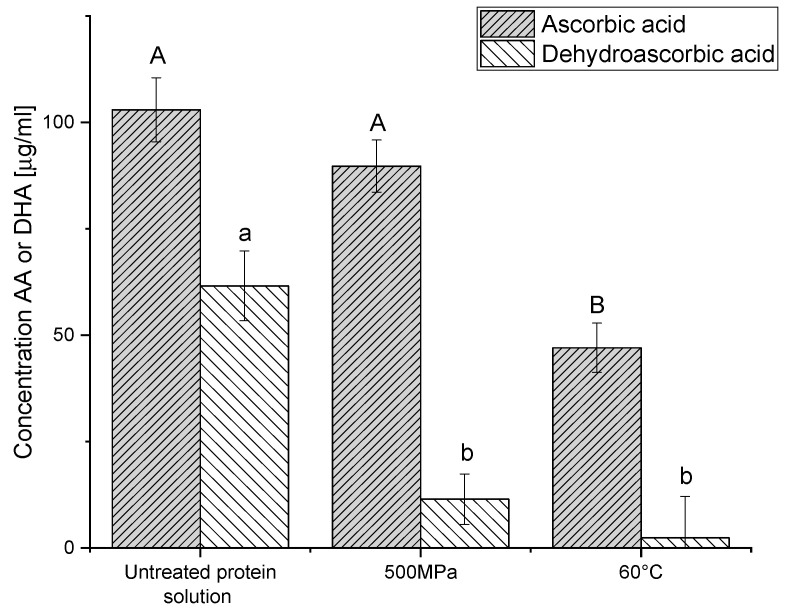
Concentration of ascorbic acid and dehydroascorbic acid in the untreated protein solutions and after gelation by pressure and heat. Different letters indicate significant differences (*p* < 0.05) for the same compound.

A possible applicative motivation to prepare protein gels by pressure and not by heat, when both treatments allow gelation, could be conservation of thermally liable compounds, either naturally present or externally added. Pressure treatment has the advantage of forming the gels at lower temperatures and transmitting pressure uniformly and quasi-instantaneously over the whole product [85], unlike thermal gelation where heating is size-dependent due to heat transfer and can even result in non-uniform gels [86]. Such differences are expected to allow better conservation of heat-liable compounds in HHP formed gels. We have verified this hypothesis by studying the retention during gelation of a model heat liable compound, Vitamin C, in gels that have a similar hardness and rheological behavior (500 MPa and 60 °C). Vitamin C is the sum of the content of two active forms, ascorbic acid (AA) and dehydroascorbic acid (DHA). These two substances are thermally liable and easily oxidized. While the oxidation of AA to DHA is reversible, DHA can be irreversibly hydrolyzed to diketogulonic acid (DGA), which is not as bioactive as vitamin C [87]. To quantify total vitamin C, DHA is reduced to AA. The DHA content can be calculated as the difference between the total vitamin C (after DHA reduction) and the AA concentrations (without reduction). It can be seen in Figure 7 that the level of AA after pressure treatment is higher than after heat treatment and is not statistically different from the level extracted from non-treated PPI solution, but for DHA, there is no statistically significant difference between treatments, and they are both lower than the levels in the untreated PPI solution. Thus, both gelation treatments decrease vitamin C content (the sum of AA and DHA), yet the decrease due to high pressure processing is lower than in the case of heat treatment. From previous works, it is known that in fruit juices and purees (after the same treatment time), heat treatment results in a loss of ascorbic acid of about 20–25% at 45 °C; above 75 °C there is a loss of 60–70%, while pressure treatment can preserve the majority of vitamin C [87].

## 4. Conclusions

Our results present that both pressure and heat can allow the formation of gels from potato protein isolate as a novel plant-based protein source. Thermally formed physical gels were obtained at both pH 3 and pH 7 with minimal gelation temperatures around 45–50 °C. Similarly, pressure, but only when reaching a sufficiently high temperature during pressurization (in the time frame of 30 min), can result in the formation of physical gels at pH 3, while at pH 7, for the studied buffer, the application of pressure resulted in an aggregation but not in stable physical gels. Our results suggested that aggregation, likely due to pH induced shifts during pressurization, prevented the formation of the ordered three-dimensional (3D) network, suggesting that for optimal pressure induced gelation the impact of pressure on pH should also be considered. The large Mw proteins are strongly involved in the network formation, while Patatin is partially released in all gelation conditions, suggesting that it is not the major contributor to the 3D network for the tested conditions. All results point towards different molecular interactions occurring in the pressure and thermal gels, even when the rheological curves are similar. The improved preservation of thermally liable compounds in the pressure-formed gels can provide an applicative advantage for the utilization of pressure for protein gel formation. Further works should also focus on the kinetics of gel formation, which was not addressed during this study and can have a major impact on both fundamental aspects and possible applications.

## Figures and Tables

**Table 1:** The slope (n) and constant (k) of the mechanical spectrum fitting to Equation (2) of the thermally and pressure induced gels.

Temp/Pressure	pH = 3		pH = 7	
	n	K [Pa]	n	K [Pa]
45 °C			1.471 ± 0.185 ^a^	1.85 ± 2.34 ^a^
50 °C	0.317 ± 0.021 ^a,D^	154.5 ± 1.3 ^a,D^	0.222 ± 0.024 ^b,E^	2167.7 ± 59.8 ^b,E^
55 °C	0.246 ± 0.020 ^b,D^	1506.6 ± 118.1 ^b,D^	0.214 ± 0.007 ^b,D^	4325.1 ± 176.1 ^c,E^
60 °C	0.185 ± 0.002 ^b,D^	21,037.8 ± 554.8 ^c,D^	0.172 ± 0.010 ^b,D^	12,078 ± 180.5 ^d,E^
90 °C	0.182 ± 0.001 ^b,D^	18,323.1 ± 696.3 ^c,D^	0.169 ± 0.007 ^b,D^	12,105.9 ± 364.7 ^d,D^
300 MPa	0.242 ± 0.020 ^b^	1932.0 ± 61.1 ^b^		
400 MPa	0.238 ± 0.043 ^b^	2624.2 ± 102.9 ^b^		
500 MPa	0.189 ± 0.005 ^b^	4698.9 ± 179.2 ^d^		

Difference in the superscript small letter represents a statistical difference between processing conditions at the same pH, while differences in the capital letter represent statistically significant differences for the same temperature at the different pH levels (*p* < 0.05, *n* = 3).
